# Investigation about the Effect of Manufacturing Parameters on the Mechanical Behaviour of Natural Fibre Nonwovens Reinforced Thermoplastic Composites

**DOI:** 10.3390/ma12162560

**Published:** 2019-08-11

**Authors:** Imen Gnaba, Peng Wang, Damien Soulat, Fatma Omrani, Manuela Ferreira, Philippe Vroman

**Affiliations:** 1 GEMTEX, ENSAIT, University of Lille, F-59056 Roubaix, France; 2 Composites Centre, AMRC with Boeing, University of Sheffield, Sheffield S60 5ZT, UK

**Keywords:** nonwoven, natural fibre composites (NFC), thermoplastic, mechanical behaviour

## Abstract

To date, nonwoven fabrics made with natural fibres and thermoplastic commingled fibres have been extensively used in the composite industry for a wide variety of applications. This paper presents an innovative study about the effect of the manufacturing parameters on the mechanical behaviour of flax/PP nonwoven reinforced composites. The mechanical properties of nonwoven fabric reinforced composites are related directly to the ones of dry nonwoven reinforcements, which depend strongly on the nonwoven manufacturing parameters, such as the needle-punching and areal densities. Consequently, the influence of these manufacturing parameters will be analysed through the tensile and flexural properties. The results demonstrated that the more areal density the nonwoven fabric has, the more the mechanical behaviour can be tested for composites. By contrast, it has a complex influence on needle-punching density on the load-strain and bending behaviours at the composite scale.

## 1. Introduction

Following recent developments in terms of lightness, safety, comfort, respect for the environment, and low energy consumption of the automobile, plastic and composite materials are being used increasingly in the automotive industry [1,2]. This transport sector shows the most significant growth in the use of composites between 2016 and 2021 (21%) [3]. In this sector, some drastic regulations have been implemented to comply with standards and European directives that promote end-of-life treatment by recycling materials [4,5]. In response, the design of mechanically recyclable composites involves the development of thermoplastic matrices associated with natural fibres as reinforcement. For these applications, hemp or flax fibres are the most frequently used, due to their highly specific mechanical properties [6,7] and moderate cost, specifically in Europe, where production areas reach 114,000 ha per year and in France where 75% of European flax fibre (2001–2008) is grown [8]. The applied fibre semi-products are raw fibres and non-woven mats, and the composites, therefore, possess moderate mechanical properties that make them well qualified for non-structural parts [9]. For example, rear-window shelves, door panels, and car roofs are used for spare-wheel covers and noise absorber panels [10]. With an Ashby approach, Shah [11] has shown that, by taking into account the costs of manufacturing, nonwovens reinforcements are more unique, especially in terms of tensile properties per unit cost. In addition, non-woven manufacturing processes allow the reintroduction of ground wastes in new nonwoven and, thus, reuse waste production, which is a drawback of nonwoven composite manufacturing [8,12]. Among manufacturing processes [13,14], three-dimensional needle-punching allows producing complex net-shape/near-net-shape preforms [15]. The design of mechanically recyclable composites involves the development of thermoplastic matrices instead of thermoset resins. The most studied thermoplastic is poly-(propylene) (PP), due to its low cost, chemical stability, and low density. There is an increasing development of structural thermoplastic biocomposites with natural fibres [16]. Bourmaud and Baley [17,18] have studied the recycling of injected PP-hemp or sisal 59 (30% wt) composites in comparison to PP-glass fibre. Due to the division of fibre 60 bundles, plant fibre composites exhibit only a slight change of the fibre aspect ratio throughout the process cycle, which induces a quasi-stability of their mechanical performances. In another recent study [19], during recycling, a material undergoes several cycles at 210 °C and remains usable. Lastly, the degradation of the bio-composites was also discussed with enhanced exposure time [20]. For all these reasons, the use of compression moulded non-woven PP/flax biocomposites is greatly appreciated in vehicle manufacturing. However, mechanical properties of these products (PP/Flax needle-punched nonwoven) at the dry scale (before compression moulding) and at the composite scales (after compression moulding) are very dependent on several parameters, which include the parameters associated with the process (such as the needle-punching density, depth of punching), the characteristics of manufactured nonwovens (such as areal density and fibre orientation distribution), and the parameters linked to the final composites (fibre volume fraction or porosity content) [21,22].

At the dry scale, and concerning the needle-punching density, Das et al. [14] report that higher punch densities result in denser fabrics with higher tensile strength, abrasion resistance, bursting strength, and tear resistance, but also results in greater amounts of fibre damage and fibre breakage. On needle-punched jute nonwovens, Maity [23] has exhibited the anisotropy of the tensile behaviour between MD/CD directions (Machine direction/Cross direction). With increasing punch density, the strength of nonwoven fabric reaches the maximum level and then falls [24,25,26,27,28,29]. Ishikawa et al. [30] have recently studied the effect of needle-punching conditions on fibre orientations of non-woven structures by X-ray computed tomography and have linked these orientations to the tensile behaviour of nonwoven. At composite scales. A large number of studies report mechanical properties obtained on flax/PP nonwoven composites. For a Flax/Epoxy nonwoven composite of 300 g/m^2^ (areal density) and with a Vf equal to 30%, Bensadoun et al. [31] have found a tensile strength of 84 MPa, a tensile failure strain of 1.49%, and a first tensile stiffness of 7.3 GPa along with a second tensile stiffness of 5.6 GPa. This decreased the strain softening. Miao et al. [32], from a needle-punched carded nonwoven, have dissociated, for a Flax/PP composite (Vf = 28.5%), the longitudinal properties (Tensile: Strength 88 MPa, Modulus: 5 GPa, Flexural: Strength 90 MPa, Modulus: 6.28 GPa) to the perpendicular properties (Tensile: Strength 33 MPa, Modulus: 2.73 GPa, Flexural: Strength 54 MPa, Modulus: 2.74 GPa). These results are in the same range as those given by Pickering et al. [33] in case of Flax nonwoven thermoplastic with a tensile modulus between 4–8 GPa and tensile strength of 40–60 MPa. With an experimental study on Flax/PP nonwovens, Giri Dev et al. [34] have reported recently that the increase in needling density led to the deterioration of mechanical properties (tensile and flexural) of composites due to fibre breakage and voids.

Few studies have been conducted regarding the evolution of the needle punching density of the isotropy ratio among these mechanical properties in the main directions (MD/CD) of nonwoven materials. A previous paper [35] associated with an experimental study conducted at the dry scale with the same materials has shown that the needle-punching density can influence the anisotropy of nonwoven fabrics. The purpose of this new paper is to analyse the mechanical behaviour of nonwoven reinforced Flax/PP by tensile and flexural tests conducted at the scale of composites after the hot moulding process. The influence of process parameters like the needle punching density as well as the modification of areal density was studied at both scales. The mechanical property results were compared with results quoted in this introduction.

## 2. Materials and Methods

### 2.1. Nonwoven Reinforcements and Composites

The tested materials represent an integral part of the final product properties. Appropriate materials need to be employed in order to manufacture composites with the required performance. In the present study, two sets of Flax/PP nonwoven fabrics were chosen to manufacture the composites. The main properties of the nonwoven reinforcements are noted in Table 1. The nonwovens in set A have the same needle-punching density and the different areal density. By contrast, the set B nonwovens have an identical areal density and the different needle-punching density.

**Figure 1:**
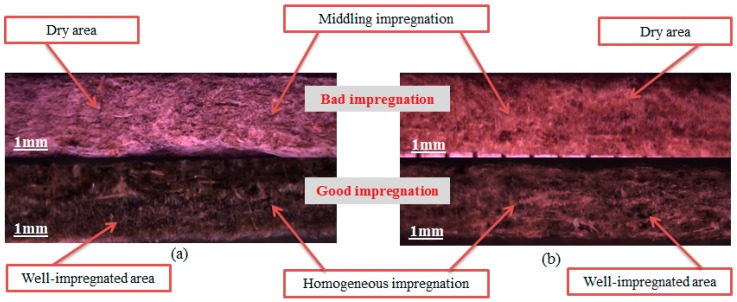
Microscopic observation on the cross-section, (**a**) A2000 nonwoven reinforced composite, and (**b**) B150 nonwoven reinforced composite.

The tested composites are named following the reference of the nonwoven reinforcement and divide to two sets (A and B sets). All the tested composites were manufactured by a hot-press moulding process (see Table 2). The influence on the mechanical properties of natural fibre reinforced thermoplastic composites during the hot-press moulding process has been investigated in the literature [36,37]. Several important process parameters such as the pressure, the temperature, and the time of the cycle were pointed out. One ply nonwoven fabric with the surface dimensions 290 × 290 mm^2^ is used. The suitable experimental protocol should be chosen not only to analyse the quality of the resin impregnation but also to control the thickness of the final composite part (around 2 mm). Figure 1 shows an example of the microscopic observation of the composite samples. The microscopic observation makes it possible to distinguish between good and bad impregnation.

The thickness and the Fibre Volume Fraction (Vf) of different nonwoven composite samples are shown in Table 2. The four composites in set B present homogenous thickness and Vf as their reinforcements have the same areal density. By contrast, it is difficult to get a similar Vf for A set nonwoven composites.

### 2.2. Mechanical Characterisation

The tensile characterisation for nonwoven composites was carried out according to the ISO 527. The crosshead speed used during the test is 2 mm/min and the sample dimensions are 250 × 50 mm^2^. Six samples for each group of composites in each direction (MD and CD) were prepared and tested. In addition, the three-point bending tests of nonwoven composites were performed according to the ISO 14125. The flexural behaviour can be characterised by the flexural strength ($\sigma_{f}$, in MPa), the flexural strain ($\varepsilon_{f}$), and the flexural modulus ($E_{f}$, in MPa) computed from Equation (1) to Equation (3). The dimensions of bending test samples are 80 × 10 mm^2^.

(1) $$\sigma_{f}=\frac{3PL}{2bh^{2}}$$

(2) $$\varepsilon_{f}=\frac{6Dh}{L^{2}}$$

(3) $$E_{f}=\frac{L^{3}m}{4bh^{2}}$$

where *P* is the failure load (in N), *L* is the length of the support span (in mm), *h* and *b* are the depth and width of the specimen (in mm), *D* is the displacement, and *m* is the slope of the bending load-displacement curve.

## 3. Results

### 3.1. Tensile Behaviour

**Figure 2:**
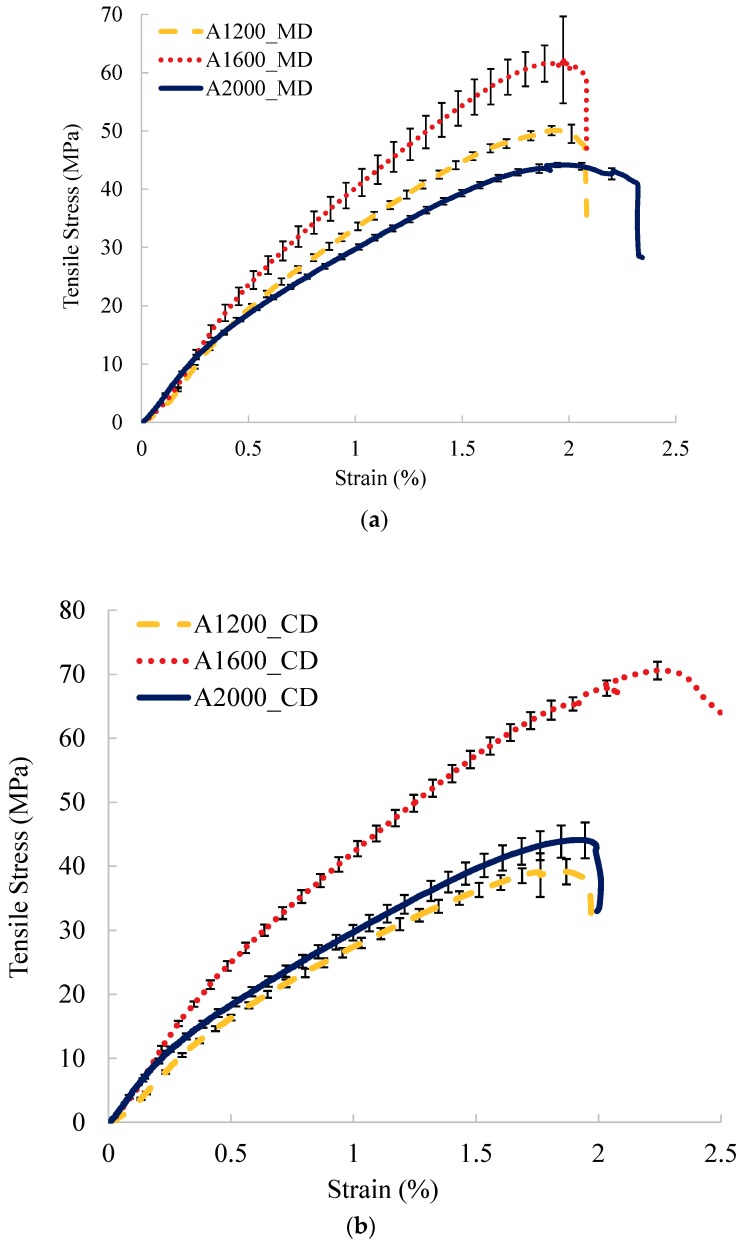

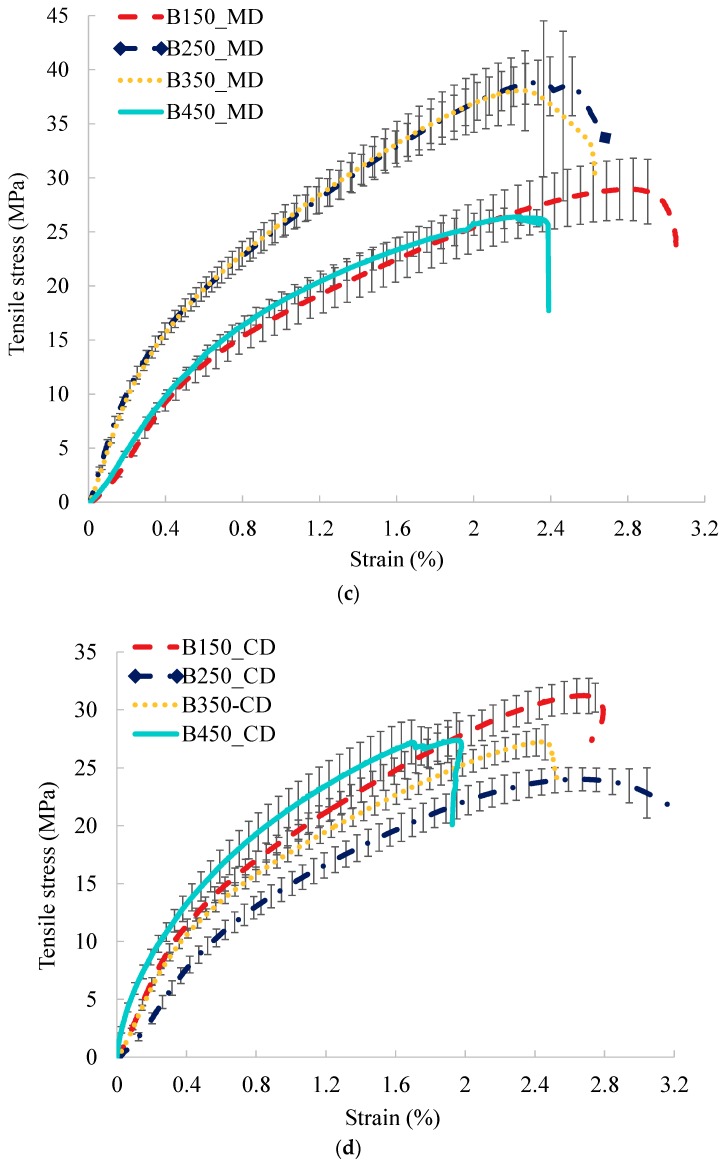
Tensile behaviour of nonwoven composites. (**a**) set A in MD, (**b**) set A in CD, (**c**) set B in MD (Machine direction), and (**d**) set B in CD (Cross direction).

Figure 2 shows the tensile stress-strain curves of A and B sets nonwoven reinforced composites. It can be noted that the tensile results of A set nonwoven composites depend strongly on the Vf presented in Table 2, which is different to the tensile results at a dry fabric scale depending on the areal density. An important Vf (48%) for the A1600 composite leads to the bigger tensile stress compared to the A1200 and A2000 composites. The A1200 and A2000 reinforced composites have similar tensile behavior since they have a quasi-same Vf, in particular in CD. As for B set nonwoven composites, the similar behaviour in CD for four samples can be noted considering the standard deviations. In MD, similar tensile behaviour can be observed for the composites reinforced by the nonwovens with the weak and high needle-punching densities (150 p/cm^2^ and 450 p/cm^2^) or with a moderate needle-punching density (250 p/cm^2^ and 350 p/cm^2^). Moreover, the more important standard deviations can be observed in MD rather than in CD.

**Figure 3:**
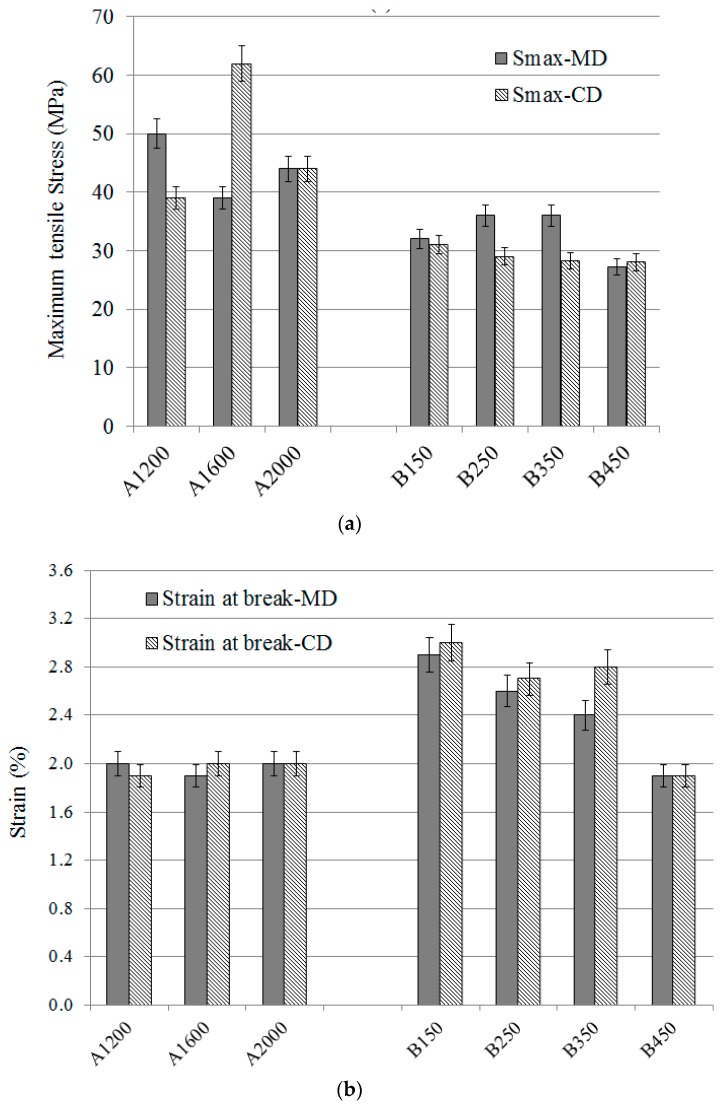
Tensile stress (**a**) and strain at break (**b**) for A and B set nonwovens reinforced composites.

From the stress-strain curves, the tensile behaviour of nonwoven composites can be characterised by the tensile strength (denoted Smax), the strain at break, and two moduli computed, respectively, in the ranges of 0–0.3% strain (denoted E1) and 0.5–1.5% strain (denoted E2). Smax and the strain at break are shown in Figure 3. Smax depends on the Vf for A set nonwovens reinforced composites. For the B set, nonwovens reinforced composites and the Smax is the quasi-same (30 MPa) in CD. By contrast, in MD, a similar Smax can be observed for the nonwovens with the weak and high needle-punching densities. The maximum strains are very similar for A set nonwovens reinforced composites in both MD and CD, which do not depend on the areal density of dry nonwovens. On the contrary, the needle-punching density influences the strain at the break at the composite scale. The maximum strain before damage decreases following the increase of the needle-punching density.

**Figure 4:**
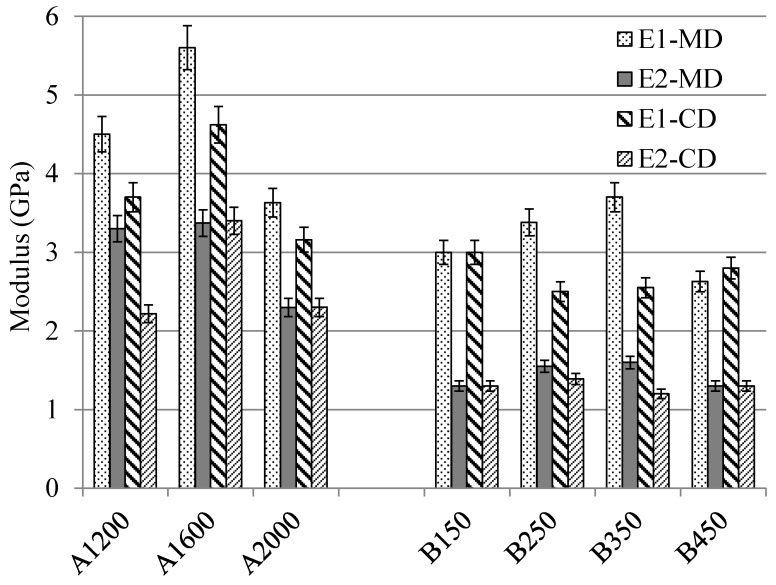
The tensile modulus of A and B nonwoven sets reinforced composites.

Figure 4 demonstrates the modulus of tensile behavior computed in each direction (MD, CD) and in each range of strain (E1 between 0% and 0.3% strain and E2 between 0.5% and 1.5% strain, as mentioned previously). Concerning the A set nonwovens reinforced composites, high Vf (48% for A1600 composite) can lead to high tensile modulus in both directions and in both ranges of the strain. The Vf is always one of the key parameters that influence the mechanical performance of the composite part. Regarding the B set nonwovens reinforced composites, they have a similar Vf, but the needle-punching density of the dry nonwoven reinforcements is different and this difference can modify the tensile modulus of the final composite part. A high tensile modulus can be obtained for the composite reinforced by the nonwovens with the moderate needle-punching densities (B250 and B350) in MD. By contrast, a homogenous tensile modulus can be observed in CD.

### 3.2. Flexural Behaviour

**Figure 5:**
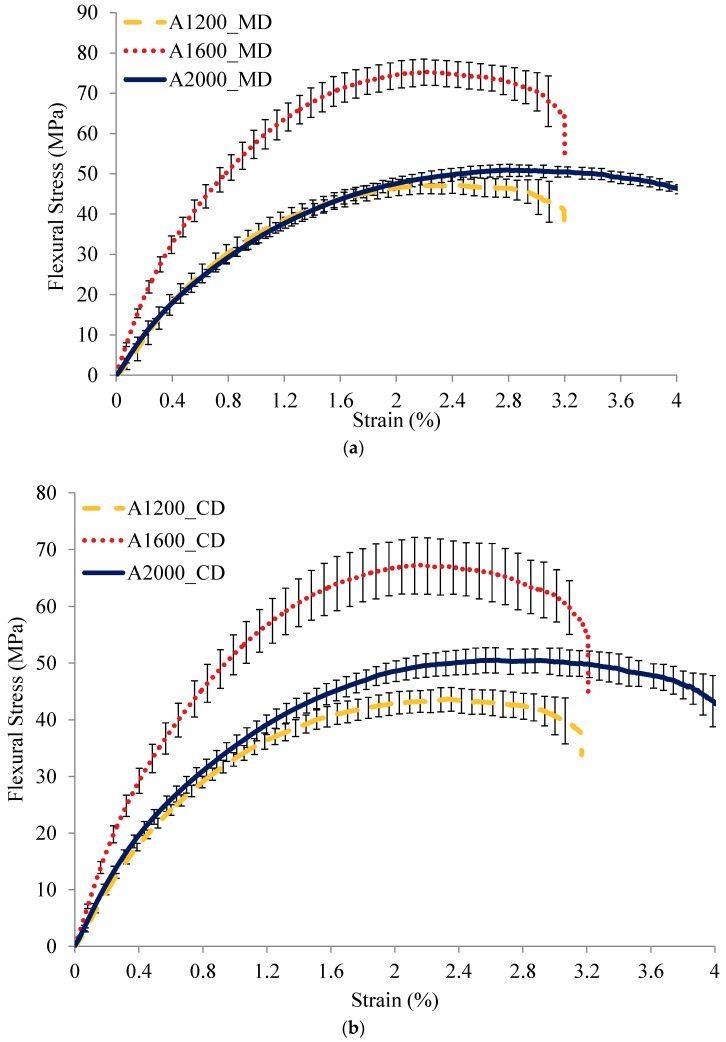

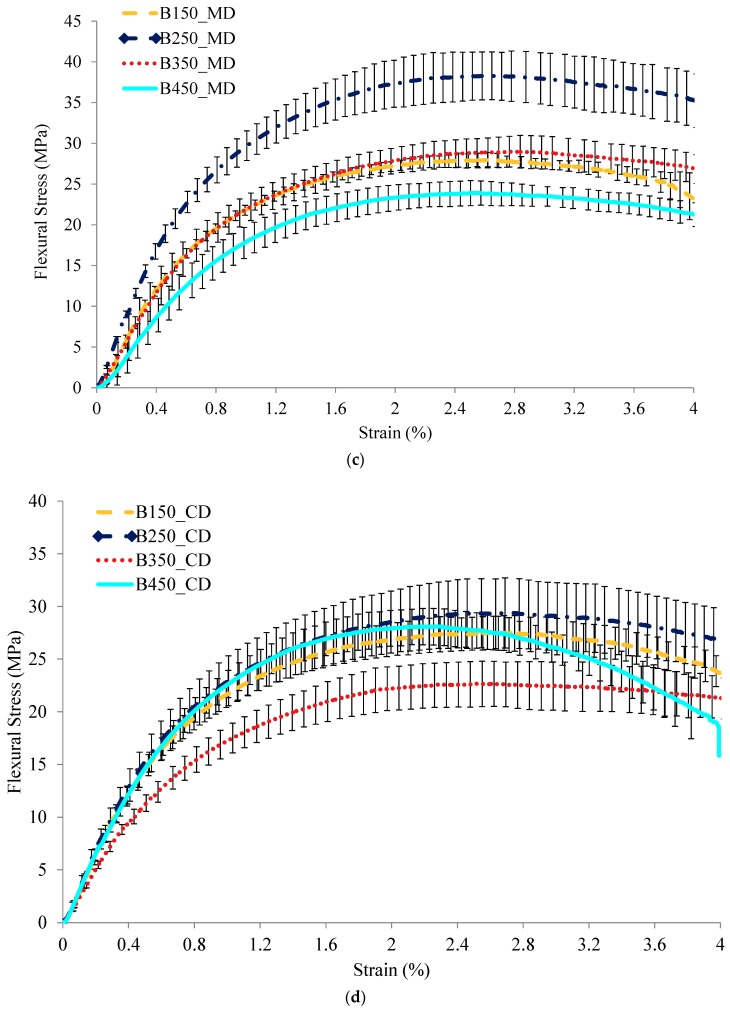
Bending behaviour of nonwoven composites. (**a**) set A in MD, (**b**) set A in CD, (**c**) set B in MD, and (**d**) set B in CD.

Figure 5 shows the flexural curves of the composites reinforced by A and B nonwoven sets. As mentioned previously, each curve is an average one of five tests. The flexural curves in both MD and CD present very non-linear progress. Regarding nonwoven set A of reinforced composites, the bending behaviour depends significantly on the Vf. The A1600 nonwoven reinforced composite has a higher bending stiffness compared to A1200 and A2000 composites. The similar Vf of A1200 and A2000 composites leads to a quite similar bending stress-strain curve. Compared to the nonwoven set A of reinforced composites, it can be observed that the clear influence of the punching density on the bending behaviour in MD of the nonwoven set B of reinforced composites. An important bending stiffness can be noted for the moderated needle-punching densities and a weak bending stiffness can be observed when a high or low needle-punching density was employed. By contrast, in CD of B set nonwovens reinforced composites, a quasi-same bending stress-strain curve is obtained. It means that the change of the needle-punching density does not affect the bending behaviour of the nonwoven reinforced composite in the cross direction.

**Figure 6:**
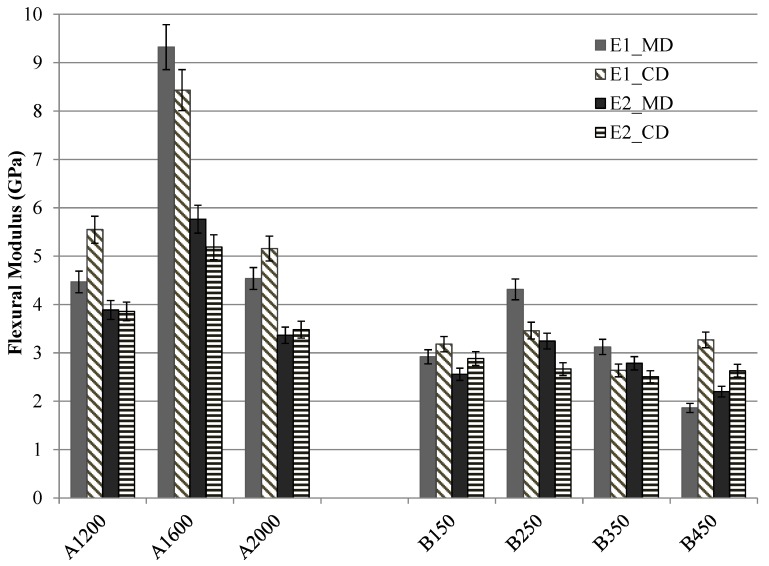
The flexural modulus of set A and set B nonwoven reinforced composites.

The analysis of the flexural modulus is figured out in Figure 6. E1 and E2 represent the flexural modulus (calculated by Equation (3)) in the range of strain between 0% and 0.3% and between 0.5% and 1.5%, respectively. It can be observed that the flexural modulus of the A set composites depends on the Vf. The higher Vf leads to the bigger modulus in two directions. By contrast, for set B composites, a higher modulus can be observed in MD when a moderated needle-punching density (B250 and B350) is used. A slight difference can be remarked in CD for four different B composites.

## 4. Discussion about the Anisotropy Phenomenon

The ratios of tensile anisotropy of nonwoven composites are shown in Table 3. As for set A composites, it can be observed that the Vf can influence the ratio of tensile stress. By contrast, the strain at break is nearly identical in CD and MD. Regarding the tensile modulus, the anisotropy phenomenon is observed mainly for E1 (the range of 0–0.3% strain on tensile curves). In general, less anisotropy phenomenon is observed at set A composites compared to set A nonwoven materials [26]. As for set B composites, the anisotropy phenomenon can be noted for the nonwoven composites reinforced by nonwoven fabrics used a moderate needle-punching density (250 and 350 p/cm^2^), which is very different from what we noted at the dry nonwoven scale [26]. 

The ratios of the flexural anisotropy at the composite scale are shown in Table 4. Regarding the set A composites, a slight anisotropy phenomenon can be noted for the first modulus E1. Apart from the tensile properties, the flexural properties for set A composites are more homogenous. As for set B composites, the B150 composite has generally good isotropy in flexural properties. By contrast, a significant anisotropy phenomenon can be reported for other composites, except for flexural strain at break. Compared to the flexural properties of dry nonwovens [26], the good homogenous can be noted at both fabric and composite scales. Since a very similar thickness was obtained in B set composites, their flexural properties depend strongly on the dry nonwoven fabric. Consequently, the similar flexural anisotropy pheromone can be remarked between the nonwoven fabrics and composites. In the MD, a weak flexural performance can be observed when a high or low needle-punching density is used and high flexural performance can be noted for the moderate needle-punching densities.

## 5. Conclusion

In the present paper, an experimental investigation has been proposed on the mechanical behaviour of flax/PP nonwoven reinforced composites. The influence of manufacturing parameters of nonwoven reinforcement on the mechanical performance of the final composite part as well as the influence of the needle-punching and the areal densities, were analysed. At the composite scale, the results of the tensile and flexural tests highlight the importance of the Vf (fibre volume fraction) directly related to the areal density of dry nonwoven reinforcement. Higher Vf leads to better mechanical properties. The needle-punching density influences the tensile and flexural behaviour in the machine direction, but not in the cross direction. In the machine direction, good tensile and flexural properties can be obtained by using the moderate needle-punching density and the weak tensile and flexural performance can be remarked on by using the high and low needle-punching densities. 

The anisotropy phenomenon can be observed at both dry fabric and composite scales. The phenomenon at the composite scale does not depend completely on the one at the dry fabric scale. In general, the areal density of nonwovens does not influence the mechanical properties of composite parts. By contrast, there is an influence of needle-punching density of dry nonwoven materials on the mechanical performance of nonwoven composites. The homogenous behaviour of mechanical properties can be observed when a high or low needle-punching density is employed.

## Figures and Tables

**Table 1:** The main properties of the nonwoven reinforcements.

Reference of Nonwoven Fabric	Set	Mass Ratio Flax/PP (%)	Flax Fibre Length (mm)	PP Fibre Length (mm)	Needle Punching Density (p/cm^2^)	Areal Density (g/m^2^)
A1200	A	50/50	60	40	50	1200 ± 50
A1600						1600 ± 50
A2000						2000 ± 60
B150	B				150	1600 ± 100
B250					250	
B350					350	
B450					450	

**Table 2:** The main parameters of the tested nonwoven composites.

Reference of Nonwoven Composite	Set	Nonwoven Reinforcement	Manufacturing Process	Thickness (mm)	Vf (%)
A1200	A	A1200	Hot-press moulding	1.4 ± 0.2	33.0 ± 3.7
A1600		A1600		1.4 ± 0.4	48.0 ± 2.0
A2000		A2000		1.8 ± 0.1	34.0 ± 2.0
B150	B	B150		1.8 ± 0.1	42.0 ± 1.5
B250		B250		2.0 ± 0.1	40.0 ± 1.9
B350		B350		2.0 ± 0.2	40.0 ± 4.0
B450		B450		2.1 ± 0.1	39.0 ± 2.0

**Table 3:** Ratios of tensile anisotropy of nonwoven composites.

Composites	Ratio CD/MD for Maximum Tensile Stress	Ratio CD/MD for Strain at Break	Ratio CD/MD for Tensile Modulus 1	Ratio CD/MD for Tensile Modulus 2
A1200	0.80	1.06	0.82	0.92
A1600	1.06	1.05	0.83	1.01
A2000	0.91	0.95	0.87	1.00
B150	0.97	1.03	1.00	1.00
B250	0.80	1.04	0.74	0.89
B350	0.79	1.17	0.70	0.82
B450	1.03	1.00	1.06	1.00

**Table 4:** Ratios of flexural anisotropy of nonwoven composites.

Reinforcements	Ratio CD/MD for Maximum Flexural Stress	Ratio CD/MD for Flexural Strain at Break	Ratio CD/MD for Flexural Modulus 1	Ratio CD/MD for Flexural Modulus 2
A1200	1.03	0.98	1.24	0.99
A1600	1.0	1.00	0.90	0.90
A2000	1.05	0.93	1.14	1.03
B150	0.99	1.02	1.09	1.13
B250	0.80	1.00	0.80	0.82
B350	0.77	0.99	0.84	0.90
B450	1.18	0.90	1.76	1.19
